# Supplementary material for: The Revival of Aztreonam in Combination with Avibactam against Metallo-β-Lactamase-Producing Gram-Negatives: A Systematic Review of In Vitro Studies and Clinical Cases
Source: Antibiotics (Basel). 2021 Aug 20;10(8):1012. doi: 10.3390/antibiotics10081012 (PMC8388901; doi:10.3390/antibiotics10081012)
Supplement: Supplementary file 1 [file antibiotics-10-01012-s001.zip › antibiotics-1317356-supplementary.pdf]

Supplementary materials

Table S1. Quality assessment of *in vitro* studies

Type of study: In vitro study(s)						
**Study**	**Domains**					
	Were the criteria for inclusion in the sample clearly defined?	Was the isolation background (Country/type of collection, study period) described in detail?	Was the antimicrobial activity measured in a valid and reliable way?	Were standard criteria used for susceptibility assessment?	Were resistance determinants other than MBLs described in detail?	Was the antimicrobial activity described in detail?
Livermore, 2011 [15]	Yes	No	Yes	Yes	No	Yes
Wang, 2014 [16]	Yes	Yes	Yes	Yes	Yes	Yes
Alm, 2015 [17]	Yes	No	Yes	Yes	Yes	Yes

Kazmierczak, 2015 [18]	Yes	Yes	Yes	Yes	No	Yes
Li, 2015 [19]	Yes	No	Yes	Yes	Yes	Yes
Vasoo, 2015 [20]	Yes	No	Yes	Yes	No	Yes
Pillar, 2016 [21]	Yes	No	Yes	Yes	No	Yes
Thomson, 2016 [22]	Yes	No	Yes	Yes	No	Yes
Karlowsky, 2017 [23]	Yes	Yes	Yes	Yes	No	Yes
Marshall, 2017 [24]	Yes	No	Yes	Yes	Yes	Yes
Wenzler, 2017 [25]	Yes	No	Yes	Yes	No	No
Zhang, 2017 [26]	Yes	Yes	Yes	Yes	No	Yes
Avery, 2018 [27]	Yes	No	Yes	Yes	Yes	No

Jayol, 2018 [28]	Yes	No	Yes	Yes	No	Yes
Sader, 2018 [29]	Yes	Yes	Yes	Yes	No	Yes
Biagi, 2019 [30]	Yes	No	Yes	Yes	Yes	Yes
Lin, 2019 [31]	Yes	Yes	Yes	Yes	No	Yes
Mikhail, 2019 [32]	Yes	No	Yes	Yes	Yes	Yes
Pragasam, 2019 [33]	Yes	No	Yes	Yes	No	Yes
Zou, 2019 [34]	Yes	Yes	Yes	Yes	No	Yes
Esposito, 2020 [35]	Yes	Yes	Yes	Yes	No	Yes
Kilic, 2020 [36]	Yes	No	Yes	Yes	No	Yes
Kim, 2020 [37]	Yes	Yes	Yes	Yes	No	Yes

Lee, 2021 [38]	Yes	No	Yes	Yes	Yes	Yes
Niu, 2020 [39]	Yes	No	Yes	Yes	No	Yes
Periasamy, 2020 [40]	Yes	Yes	Yes	Yes	No	Yes
Wei, 2020 [41]	Yes	Yes	Yes	Yes	No	Yes
Yang, 2020 [42]	Yes	Yes	Yes	Yes	No	Yes
Zhang, 2020 [43]	Yes	Yes	Yes	Yes	No	Yes
Zou, 2020 [44]	Yes	Yes	Yes	Yes	No	Yes
Bhatnagar, 2021 [45]	Yes	No	Yes	Yes	No	Yes
Chang, 2021 [46]	Yes	Yes	Yes	Yes	No	Yes
Falcone, 2021 [47]	Yes	Yes	Yes	Yes	No	No

Lin, 2021<br>[48]	Yes	Yes	Yes	Yes	No	Yes
Maraki, 2021<br>[49]	Yes	Yes	Yes	Yes	No	Yes

Table S2. Quality assessment of clinical studies: case(s) report

Type of study: case(s) report. Potential responses: yes/no/unclear/not applicable									
**Study**	**Domains**								
	Were patient's demographic characteristics clearly described?	Was the patient's history clearly described and presented as a timeline?	Was the current clinical condition of the patient on presentation clearly described?	Were diagnostic tests or assessment methods and the results clearly described?	Was the intervention(s) or treatment procedure(s) clearly described?	Was the post-intervention clinical condition clearly described?	Were adverse events (harms) or unanticipated events identified and described?	Does the case report provide takeaway lessons?	Any comment
Mojica, 2016 [50]	Yes	Yes	Yes	Yes	Yes	Yes	No	Yes	
Davido, 2017 [51]	Yes	Yes	Yes	Yes	Yes	Yes	No	Yes	
Mittal, 2018 [52]	Yes	Yes	Yes	Yes	Yes	Yes	Yes	Yes	
Emeraud, 2019 [54]	Yes	Yes	Yes	Yes	Yes	Yes	No	Yes	
Hobson, 2019 [55]	Yes	Yes	Yes	Yes	Yes	Yes	No	Yes	

Shah, 2019 [56]	Yes	Yes	Yes	Yes	Yes	Yes	No	Yes	
Stewart, 2019 [57]	Yes	Yes	Yes	Yes	Yes	Yes	Yes	Yes	
Bencherit, 2020 [58]	Yes	Yes	Yes	Yes	Yes	Yes	No	Yes	
Alghoribi, 2021 [59]	Yes	Yes	Yes	Yes	Yes	Yes	No	Yes	
Bocanegra-Ibarias, 2021 [60]	Yes	Yes	Yes	Yes	Yes	Yes	No	Yes	
Cowart, 2021 [62]	Yes	Yes	Yes	Yes	Yes	Yes	No	Yes	
Perrotta, 2021 [63]	Yes	Yes	Yes	Yes	Yes	Yes	No	Yes	
Sieswerda, 2021 [64]	Yes	Yes	Yes	Yes	Yes	Yes	Yes	Yes	
Yasmin 2021 [65]	Yes	Yes	Yes	Yes	Yes	Yes	No	Yes	

Table S3. Quality assessment of clinical studies: case series

Type of study: case series. Potential responses: yes/no/unclear/not applicable										
**Study**	**Domains**									
	Were there clear criteria for inclusion in the case series?	Was the condition measured in a standard, reliable way for all participants included in the case series?	Were valid methods used for identification of the condition for all participants included in the case series?	Did the case series have consecutive inclusion of participants?	Did the case series have complete inclusion of participants?	Was there clear reporting of the demographics of the participants in the study?	Was there clear reporting of clinical information of the participants?	Were the outcomes or follow up results of cases clearly reported?	Was there clear reporting of the presenting site(s)/clinic(s) demographic information?	Was statistical analysis appropriate?
Shaw, 2018 [53]	Yes	Yes	Yes	No	Unclear	Yes	Yes	Yes	Yes	Not applicable
Cairns, 2021 [61]	Yes	Yes	Yes	No	Unclear	Yes	Yes	Yes	Yes	Not applicable

Table S4. Quality assessment of clinical studies: cohort study

Type of study: case series. Potential responses: yes/no/unclear/not applicable.											
**Study**	**Domains**										
	Were the two groups similar and recruited from the same population?	Were the exposures measured similarly to assign people to both exposed and unexposed groups?	Was the exposure measured in a valid and reliable way?	Were confounding factors identified?	Were strategies to deal with confounding factors stated?	Were the groups/ participants free of the outcome at the start of the study (or at the moment of exposure)?	Were the outcomes measured in a valid and reliable way?	Was the follow up time reported and sufficient to be long enough for outcomes to occur?	Was follow up complete, and if not, were the reasons to loss to follow up described and explored?	Were strategies to address incomplete follow up utilized?	Was appropriate statistical analysis used?
Falcone, 2020 [4]	Yes	Yes	Yes	Yes	Yes	Yes	Yes	Yes	Yes	Yes	Yes

Falcone, 2021 [47]	Yes	Yes	Yes	Yes	Yes	Yes	Yes	Yes	Yes	Yes	Yes

Figure S1. PRISMA 2020 checklist

Section and Topic	Item #	Checklist item	Location where item is reported
**TITLE**			
Title	1	Identify the report as a systematic review.	Pag.1, Sec. 1
**ABSTRACT**			
Abstract	2	See the PRISMA 2020 for Abstracts checklist.	Pag.1, Sec. 1
**INTRODUCTION**			
Rationale	3	Describe the rationale for the review in the context of existing knowledge.	Pag. 2/3, Sec. 1
Objectives	4	Provide an explicit statement of the objective(s) or question(s) the review addresses.	Pag. 2/3, Sec. 1
**METHODS**			
Eligibility criteria	5	Specify the inclusion and exclusion criteria for the review and how studies were grouped for the syntheses.	Pag. 34/35, Sec. 4
Information sources	6	Specify all databases, registers, websites, organisations, reference lists and other sources searched or consulted to identify studies. Specify the date when each source was last searched or consulted.	Pag. 34/35, Sec. 4
Search strategy	7	Present the full search strategies for all databases, registers and websites, including any filters and limits used.	Pag. 34/35, Sec. 4
Selection process	8	Specify the methods used to decide whether a study met the inclusion criteria of the review, including how many reviewers screened each	Pag. 35, Sec. 4

Section and Topic	Item #	Checklist item	Location where item is reported
		record and each report retrieved, whether they worked independently, and if applicable, details of automation tools used in the process.	
Data collection process	9	Specify the methods used to collect data from reports, including how many reviewers collected data from each report, whether they worked independently, any processes for obtaining or confirming data from study investigators, and if applicable, details of automation tools used in the process.	Pag. 35, Sec. 4
Data items	10a	List and define all outcomes for which data were sought. Specify whether all results that were compatible with each outcome domain in each study were sought (e.g. for all measures, time points, analyses), and if not, the methods used to decide which results to collect.	Pag. 35, Sec. 4
	10b	List and define all other variables for which data were sought (e.g. participant and intervention characteristics, funding sources). Describe any assumptions made about any missing or unclear information.	Pag. 35, Sec. 4
Study risk of bias assessment	11	Specify the methods used to assess risk of bias in the included studies, including details of the tool(s) used, how many reviewers assessed each study and whether they worked independently, and if applicable, details of automation tools used in the process.	Pag. 35/36, Sec. 4
Effect measures	12	Specify for each outcome the effect measure(s) (e.g. risk ratio, mean difference) used in the synthesis or presentation of results.	Pag. 35/36, Sec. 4
Synthesis methods	13a	Describe the processes used to decide which studies were eligible for each synthesis (e.g. tabulating the study intervention characteristics and comparing against the planned groups for each synthesis (item #5)).	Pag. 35/36, Sec. 4
	13b	Describe any methods required to prepare the data for presentation or synthesis, such as handling of missing summary statistics, or data conversions.	Pag. 35/36, Sec. 4
	13c	Describe any methods used to tabulate or visually display results of individual studies and syntheses.	Pag. 35/36, Sec. 4
	13d	Describe any methods used to synthesize results and provide a rationale for the choice(s). If meta-analysis was performed, describe the model(s), method(s) to identify the presence and extent of statistical heterogeneity, and software package(s) used.	Pag. 35/36, Sec. 4
	13e	Describe any methods used to explore possible causes of heterogeneity among study results (e.g. subgroup analysis, meta-regression).	Pag. 35/36, Sec. 4
	13f	Describe any sensitivity analyses conducted to assess robustness of the synthesized results.	Pag. 35/36, Sec. 4

Section and Topic	Item #	Checklist item	Location where item is reported
Reporting bias assessment	14	Describe any methods used to assess risk of bias due to missing results in a synthesis (arising from reporting biases).	Pag. 35/36, Sec. 4
Certainty assessment	15	Describe any methods used to assess certainty (or confidence) in the body of evidence for an outcome.	Pag. 35/36, Sec. 4
**RESULTS**			
Study selection	16a	Describe the results of the search and selection process, from the number of records identified in the search to the number of studies included in the review, ideally using a flow diagram.	Pag. 4, Sec. 2
	16b	Cite studies that might appear to meet the inclusion criteria, but which were excluded, and explain why they were excluded.	Pag. 4/5, Sec. 2
Study characteristics	17	Cite each included study and present its characteristics.	Pag. 4-32, Sec. 2
Risk of bias in studies	18	Present assessments of risk of bias for each included study.	Suppl. files
Results of individual studies	19	For all outcomes, present, for each study: (a) summary statistics for each group (where appropriate) and (b) an effect estimate and its precision (e.g. confidence/credible interval), ideally using structured tables or plots.	Pag. 5, Sec. 2
Results of syntheses	20a	For each synthesis, briefly summarise the characteristics and risk of bias among contributing studies.	Pag. 5, 21, Sec. 2
	20b	Present results of all statistical syntheses conducted. If meta-analysis was done, present for each the summary estimate and its precision (e.g. confidence/credible interval) and measures of statistical heterogeneity. If comparing groups, describe the direction of the effect.	Pag. 32, Sec. 2
	20c	Present results of all investigations of possible causes of heterogeneity among study results.	Pag. 32, Sec. 2
	20d	Present results of all sensitivity analyses conducted to assess the robustness of the synthesized results.	NA
Reporting biases	21	Present assessments of risk of bias due to missing results (arising from reporting biases) for each synthesis assessed.	NA

Section and Topic	Item #	Checklist item	Location where item is reported
Certainty of evidence	22	Present assessments of certainty (or confidence) in the body of evidence for each outcome assessed.	Pag. 4, Sec. 2
**DISCUSSION**			
Discussion	23a	Provide a general interpretation of the results in the context of other evidence.	Pag. 33-35, Sec. 3
	23b	Discuss any limitations of the evidence included in the review.	Pag. 33-35, Sec. 3
	23c	Discuss any limitations of the review processes used.	Pag. 33-35, Sec. 3
	23d	Discuss implications of the results for practice, policy, and future research.	Pag. 33-35, Sec. 3
**OTHER INFORMATION**			
Registration and protocol	24a	Provide registration information for the review, including register name and registration number, or state that the review was not registered.	Pag. 33, Sec. 4
	24b	Indicate where the review protocol can be accessed, or state that a protocol was not prepared.	Pag. 33, Sec. 4
	24c	Describe and explain any amendments to information provided at registration or in the protocol.	NA
Support	25	Describe sources of financial or non-financial support for the review, and the role of the funders or sponsors in the review.	Pag. 35, Sec. 5
Competing interests	26	Declare any competing interests of review authors.	Pag. 35, Sec. 5
Availability of data, code and other materials	27	Report which of the following are publicly available and where they can be found: template data collection forms; data extracted from included studies; data used for all analyses; analytic code; any other materials used in the review.	NA
